# DNA, statistics and the law: a cross-disciplinary approach to forensic inference

**DOI:** 10.3389/fgene.2014.00136

**Published:** 2014-05-15

**Authors:** Alex Biedermann, Joëlle Vuille, Franco Taroni

**Affiliations:** ^1^Faculty of Law, Criminal Justice and Public Administration, School of Criminal Justice, Institute of Forensic Science, University of LausanneLausanne, Switzerland; ^2^Department of Criminology, Law and Society, School of Social Ecology, University of CaliforniaIrvine, CA, USA

**Keywords:** forensic DNA profiling, interpretation, probability theory, commercialization, DNA transfer, low-template DNA analysis, forensic molecular biology, bacterial DNA

The use of results of DNA analyses in the legal process is a highly ambivalent topic. On the one hand, scientists have never been in a better position to analyse biological matter of various natures, even in limited quantities and degraded conditions. On the other hand, the increasing amounts of scientific data that can be generated through modern analytical processes do not necessarily imply that evaluative questions that arise in the legal context are given more satisfactory answers. A fundamental question that has accompanied DNA analyses since the early days of their use in the legal process thus remains: how do we handle the challenges presented to us by the use of contemporary scientific and technological developments in the field of law? Under the general theme “DNA, statistics and the law,” the collection of articles in this Frontiers Research Topic pursues the goal of investigating this question from an interdisciplinary perspective, and with an emphasis on both current and future challenges.

As pointed out by Gunn et al. (2014) and Leake (2013), the forensic interest in DNA goes well beyond the standard approaches to DNA profiling that represent the current state-of-the-art in many contemporary legal systems, and this raises questions as to how new forms of data ought to be dealt with in an operational perspective (Milot et al., 2013). Although these frontiers topics clarify the extent to which there is room for exciting future research in this area, it should not distract us from the fact that even in the current state of forensic practice, there are hurdles and pressing topics that ask for efficient answers. Controversies over legal cases, such as the Perugia case (Vecchiotti and Zoppis, 2013), reveal that the field is still facing difficulties in setting the meaning of DNA profiling results appropriately into context (Champod, 2013; McKenna, 2013). One might be tempted to conclude that this is an issue that is confined to (and could thus be resolved within) the intersection between forensic science and the law. This perspective might, however, fall short of further dimensions, such as commercialization (Jackson, 2013). The publication of opinion pieces on this topic helps raise awareness on this topic and address some of this deficit.

On a methodological account, the field of statistics is often invoked as a remedy to deal with evaluative questions and many discussants tend to emphasize its traditional facet concerned with data processing. The case of statistics is more general, however, because it is a branch that involves an additional characterizing feature: reasoning coherently in the face of uncertainty (known in the context as *forensic inference*), using probability theory. Indeed, existing literature abounds in rigorous and coherent approaches to cope with intricate evaluative questions (Biedermann, 2013; Juchli, 2013) of the kind that are also encountered in connection with forensic DNA. It is with some frustration, however, that we note that discussions surrounding evaluative questions, using probability, are still fraught with problems that have debates for a very long time. Prior probabilities are one example for this (Thompson et al., 2013).

In summary, the contributions in this Research Topic convince us that the extension of technical frontiers should also be accompanied by conceptual developments and understandings. Indeed, during personal discussions with the Topic Editors, one reviewer (Sheila Willis, Eolaíocht Fhóiréinseach Éireann, Forensic Science Laboratory, Ireland) raised cultural understandings as a further relevant factor: “I think the problem is much deeper. The use of matching DNA as a heuristic for a definite link between person and place is embedded in the minds of scientists as well as jurors in spite of the scholarship to the contrary. The discriminating power of DNA has had a paradoxical effect in the development of forensic science. On one hand it prompted forensic science to be valued and used in a very widespread manner but on the other hand it promoted the commodisation of forensic science with the belief that the test result is all-important and the context irrelevant. This latter view prompts the approach that the test can be produced anywhere and loses sight for the need of the very evaluation (…). It is vital that we address this. It is mixed with the commercialization issues but to focus too much on that aspect is to ignore the wider issues that also need to be addressed by: the publication of high profile cases where this approach has unfortunate consequences; increased education; critical mass of scientific opinion in favor of the approach argued for (…).”

We cannot but agree and hope that the collected papers in this Research Topic will be of interest to both scientists and other participants in the legal process. We thank all contributors and distinguished reviewers for their efforts to make this original collection timely and highly useful.

## Conflict of interest statement

The authors declare that the research was conducted in the absence of any commercial or financial relationships that could be construed as a potential conflict of interest.
